# Real-World Safety Profile of Proton Pump Inhibitors in Infants as Reported in the FDA Adverse Event Reporting System (FAERS): Tiny Tummies, Key Decisions

**DOI:** 10.3390/ph18050730

**Published:** 2025-05-16

**Authors:** Hülya Tezel Yalçın, Nadir Yalçın, Karel Allegaert

**Affiliations:** 1 Department of Pharmaceutical Toxicology, Faculty of Pharmacy, Hacettepe University, 06100 Ankara, Türkiye; hulya.tezel@hacettepe.edu.tr; 2 Department of Clinical Pharmacy, Faculty of Pharmacy, Hacettepe University, 06100 Ankara, Türkiye; nadir.yalcin@hacettepe.edu.tr; 3 Clinical Pharmacology and Pharmacotherapy, Department of Pharmaceutical and Pharmacological Sciences, KU Leuven, 3000 Leuven, Belgium; 4 Department of Development and Regeneration, KU Leuven, 3000 Leuven, Belgium; 5 Department of Hospital Pharmacy, Erasmus MC, 3015 GD Rotterdam, The Netherlands

**Keywords:** drug safety, adverse drug event, medication error, neonates, infants, acid reducers

## Abstract

**Background**: Proton pump inhibitors (PPIs) are widely used for gastric acid suppression, yet their efficacy and safety in neonates and infants remain unclear. While esomeprazole is the only Food and Drug Administration (FDA)-approved PPI for neonates and infants under 1 year of age, other PPIs are also frequently prescribed. **Objectives**: This study utilizes FDA Adverse Event Reporting System (FAERS) data to evaluate potential adverse drug events (ADEs) of PPIs, providing crucial real-world insights into their safety in this vulnerable population. **Methods**: This observational cross-sectional study was conducted using an individual case safety report (ICSR) database. Only reports in neonates or infants receiving omeprazole, pantoprazole, lansoprazole, rabeprazole, or esomeprazole monotherapy were evaluated. The most frequently prescribed PPI, the most common indication, the most reported ADE, the seriousness of AEs, and the countries reporting the highest ADE number were analyzed using 2D disproportionality analyses (e.g., reporting odds ratio (RORs)). **Results**: A total of 464 patients were included; 323 (69.6%) of them were stated as serious and 15 (3.2%) of them were stated as time-related to mortality. Most of the ADEs were reported for lansoprazole (45.9%). The most reported PPI-associated ADE was vomiting (8.8%). According to the RORs analysis, vomiting associated with PPI monotherapy was more likely to occur (RORs: 2.88, 95% CI: 2.09–3.96), which is followed by diarrhea, hypertrichosis, choking, and erythema. Additionally, medication errors were reported in 50 (10.8%) patients. **Conclusions**: ICSR databases are valuable pharmacovigilance tools. The absence of access to a causality assessment is a limitation since it limits its ability to confirm whether the ADEs are truly caused by the suspected drug, mitigated using RORs analysis. Integrating neonatal-specific algorithms could enhance drug safety evaluations, strengthen evidence-based decision-making, and improve risk–benefit assessments in neonates and infants.

## 1. Introduction

Gastroesophageal reflux (GER) is a common condition in the pediatric population, affecting approximately 50% of infants under three months of age [1]. While often a self-limiting physiological phenomenon that resolves with maturation, GER can, in some instances, progress into gastroesophageal reflux disease (GERD), leading to more severe symptoms and complications [2]. GERD is characterized by symptoms such as esophageal inflammation, mucosal damage, and a decline in the child’s general well-being, often requiring medical intervention [3]. Distinguishing between GER and GERD in infants can be particularly challenging, as both conditions present with similar manifestations, including regurgitation and irritability. However, GERD can lead to more persistent and distressing symptoms, necessitating therapeutic measures [3,4]. The mechanistic claimed link between reflux and apnea as a typical neonatal event, most common in neonates, resulted in common prescription of these drugs, while the temporal relationship has been rejected in the meantime [5,6].

Proton pump inhibitors (PPIs: omeprazole, esomeprazole, lansoprazole, dexlansoprazole, and rabeprazole) are among the most widely prescribed medications for the treatment of GERD [7]. These drugs specifically target and inhibit the gastric proton pump, which is the enzyme responsible for the final step in gastric acid production. The proton pump, or hydrogen–potassium ATPase, is located in the parietal cells of the stomach lining and plays a crucial role in the secretion of hydrochloric acid [2]. By irreversibly binding to this enzyme, PPIs reduce both the basal and stimulated secretion of gastric acid, leading to a significant decrease in acid reflux and associated symptoms [7]. It should be considered that the clearance of PPIs in infants may vary due to CYP2C19 polymorphisms, potentially affecting their efficacy and safety [8]. Despite their effectiveness in reducing gastric acid secretion, the use of PPIs in infants remains controversial, with clinical guidelines suggesting their use only after first-line and second-line non-pharmacological treatments have failed. These strategies typically include modifications to feeding practices, such as thickening agents, as well as the elimination of cow’s milk proteins from the diet in cases where a cow’s milk protein allergy is suspected [9].

While PPIs are well established as the primary treatment for GERD in older children, their application in infants, particularly those under one year of age, is less clearly defined [10]. Episodes of regurgitation following feeding are frequent because of the substantial amount (150–180 mL/kg/day) of milk needed for healthy growth during infancy [11]. However, considering that breastfeeding is recommended for the first 6 months, additional foods after 6 months may be another predisposing factor. Due to low gastric acid production, and frequent milk uptake, the stomach content during infancy primarily has a pH > 4. However, it should not be forgotten that nutrient-enriched formulas can be given to preterms and infants older than 6 months, and this may affect gastric pH. The reflux content during infancy is therefore mostly non-acidic or only slightly acidic. The effectiveness of PPIs is limited because weakly acidic volume reflux, rather than acid reflux, is more common in newborns [12]. Esomeprazole is currently the only PPI approved by the FDA for the short-term treatment (up to six weeks) of erosive esophagitis caused by acid-mediated GERD in infants aged one month to less than one year. Pantoprazole is the only PPI approved for pediatric use that is not approved for use in children younger than 5 years old. Lansoprazole is approved for use in children 1 to 11 years of age at doses of 15 mg/day for those weighing ≤ 30 kg and at 30 mg/day for those weighing > 30 kg [10]. The increasing use of PPIs in young children across high-income countries, including France, New Zealand, Denmark, Norway, and Sweden, has rightfully sparked concerns regarding their long-term safety [13].

Studies have shown that while PPIs are effective in managing GERD, they are associated with a number of short-term side effects, including headaches, nausea, diarrhea, and constipation [3,14]. The chronic use of PPIs in children has also been linked to an increased susceptibility to infections, particularly in the gastrointestinal and respiratory systems, due to the suppression of gastric acid, which is vital for the defense against pathogens [3]. Furthermore, the long-term suppression of gastric acid may disrupt the delicate balance of the gut microbiome and alter immune system functioning [15]. These mechanisms raise important questions about the broader impact of PPI therapy on the developing pediatric immune and gastrointestinal system. In addition to infections, the prolonged use of PPIs in pediatric populations is also associated with a range of potential adverse effects, such as the increased risk of bone fractures, kidney injury, allergic reactions, asthma, inflammatory bowel disease, obesity, nutrient deficiencies (vitamin B12, iron, etc.), anxiety, and depression [16,17,18,19]. Specifically in preterms, there is a clear association between PPI use and the subsequent development of necrotizing enterocolitis [20].

A well-structured pharmacovigilance (PV) system is essential for ensuring the safe use of medications and safeguarding public health. Various online PV databases, such as the U.S. Food and Drug Administration Adverse Event Reporting System (FAERS), EudraVigilance, and the Database of Adverse Event Notifications (DAEN), serve as critical resources by compiling adverse event reports, thereby facilitating the detection and assessment of potential drug-related harms [21].

In this study, we aimed to understand the adverse effects and safety of proton pump inhibitors (PPIs) in vulnerable neonatal and infant populations using the real-world pharmacovigilance data available in the FAERS database.

## 2. Results

**Figure 1:**
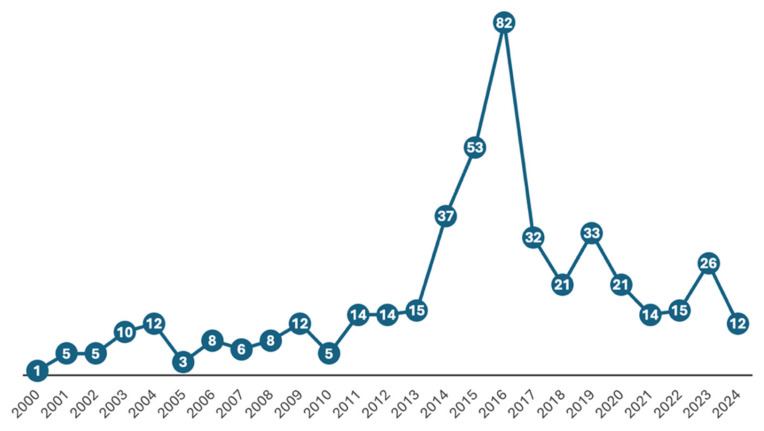
Reporting trends over the years.

After extracting the raw data, there were 1256 patients under the age of 2. Because in 70 (5.6%) patients neither their age nor weight information were available, they were excluded from the analysis. Since we focused only on patients receiving PPI treatment as monotherapy, patients receiving PPI and other pharmacotherapy, irrespective of the indication (60.9%), were also excluded. The final number of patients included in this study was 464 (36.9%) (Supplementary Materials). The reporting trends in the years between 30 June 2000 and 31 December 2024 are shown in Figure 1.

**Figure 2:**
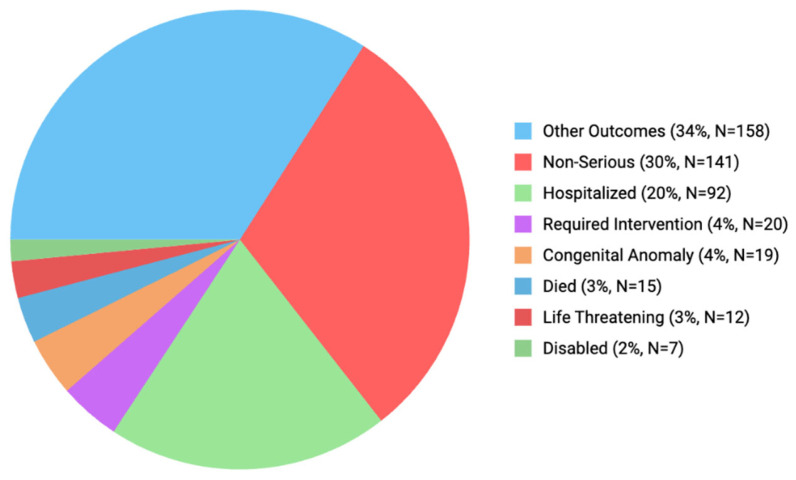
Outcomes according to the ADEs.

The outcomes according to the ADEs are classified in Figure 2. Also, 323 (69.6%) of them were stated as serious and 15 (3.2%) of these serious events were stated as death cases. Among the case reports in which death was reported, omeprazole accounted for 66.67%, lansoprazole for 23.81%, and esomeprazole for 9.52% of the cases. The age distribution of these fatal cases was as follows: 28.57% were under 1 month old, 28.57% were between 1 and 6 months old, 9.52% were between 6 months and 1 year old, 4.76% were between 1 and 2 years old, and 28.57% were 2 years old. Although the medical events observed in the patients are displayed in the database, there is no specific information available regarding the cause of death. The most frequent outcome is stated as “other outcomes” (34.1%). This is followed by “non-serious” (30.4%), “hospitalized” (19.8%), and “required intervention” (4.3%) outcomes.

**Figure 3:**
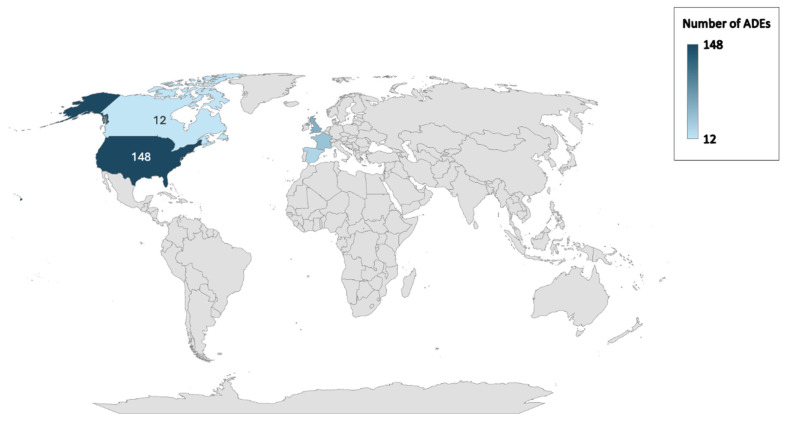
Number of ADE reports by countries.

The distribution of adverse event reports by countries is also analyzed (Figure 3). Most reports are from the United States of America (31.9%), followed by Great Britain (12.7%), France (8.83%), Spain (5.4%), and Canada (2.58%).

**Figure 4:**
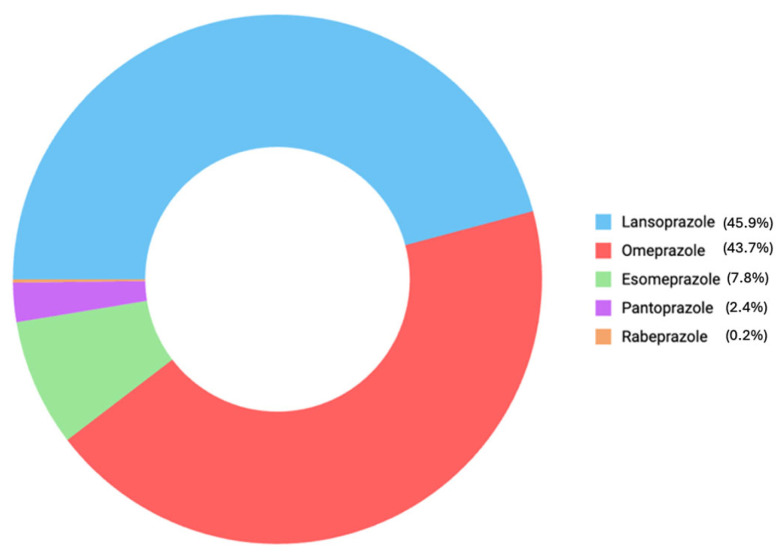
The distribution of reported ADEs for each PPI.

The distribution of reported ADEs for each PPI is shown in Figure 4. Most of the ADEs were reported for lansoprazole (45.9%). This is followed by omeprazole (43.7%), esomeprazole (7.8%), pantoprazole (2.4%), and rabeprazole (0.2%).

**Figure 5:**
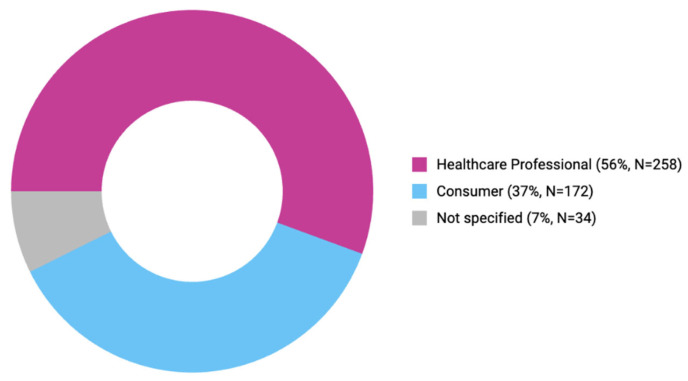
Reporter types in the FAERS database.

The reporter types in the FAERS database are given in Figure 5. According to the retrieved data, most of the ADEs were reported by healthcare professionals (55.6%), while 37.1% of the reports were submitted by consumers. In 7.3% of the ADEs, the reporter type was not specified.

**Figure 6:**
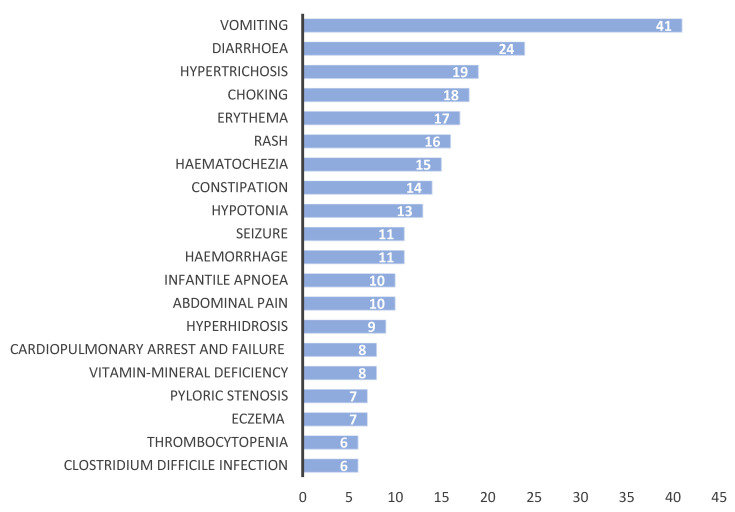
The number of reported ADEs for PPIs.

A detailed distribution of reported ADEs for PPIs is given in Figure 6. The most reported PPI-associated adverse event was vomiting (8.8%). This is followed by diarrhea (5.2%), hypertrichosis (4.1%), choking (3.9%), and erythema (3.7%). According to the RORs analysis, vomiting associated with PPI monotherapy was more likely to occur (RORs: 2.88, 95% CI: 2.09–3.96), followed by diarrhea (RORs: 2.98, 95% CI: 1.98–4.50), hypertrichosis (RORs: 79.55, 95% CI: 48.73–129.86), choking (RORs: 28.39, 95% CI: 17.52–46), and erythema (RORs: 7.30, 95% CI: 4.48–11.88). A confidence interval greater than 1 suggests a statistically significant association (*p* < 0.05).

In addition to the ADEs, additional detailed information (medication error, wrong technique in product use, off-label use, accidental exposure, etc.) was also reported. The detailed information is given in Table 1.

## 3. Discussion

GER is common during infancy. It normally disappears by the age of 1 year and typically does not require further diagnostics or special care. Sometimes it can develop into GERD when the symptoms become severe enough to require medical intervention [22]. Due to the different diagnostic criteria, it is challenging to determine the prevalence of GERD in the infant population. Approximately 25.5% of infants between the ages of 0 and 1 month have GERD, which drops to 1.1 to 1.6% by the time they are 1 year of age. About 50% of newborns under three months old suffer from GERD [3]. The most popular and widely used medications for treating GERD are PPIs.

However, and based on systematic reviews, the National Institute for Health and Care Excellence (NICE), North American Society for Pediatric Gastroenterology, Hepatology, and Nutrition (NASPGHAN), and the European Society for Pediatric Gastroenterology, Hepatology, and Nutrition (ESPGHAN) do not recommend using acid-suppressing medications as a first-line treatment for GER; instead, they suggest straightforward, low-cost therapies like slight feed changes, upright posture after meals, or thickening agents (alginate, etc.) to treat GER [22,23].

Considering the FAERS data from the past 20 years in neonates and infants, the use of PPI monotherapy exhibited an increasing trend until 2016, followed by a declining trend from 2016 to the present. Given that FDA-approved indications for PPIs in this vulnerable population are highly restricted, their prescription should be approached with caution. Based on ROR analyses conducted for the top five most frequently observed ADEs, vomiting was found to be 2.88 times, diarrhea 2.98 times, hypertrichosis 79.55 times, choking 28.39 times, and erythema 7.30 times more frequently associated with PPI monotherapy.

### 3.1. PPI Use Trends Worldwide

Illueca et al. (2014) reported that PPIs are frequently prescribed for newborns and infants in the U.S. [24]. De Bruyne et al. (2014) found that PPI use has increased significantly in children in Belgium, but PPI use was not proportional to the prevalence of GERD in children [25]. O’Reilly et al. (2020) showed that PPI prescriptions for children under one year old increased significantly over a ten-year period in Ireland [26]. Lyamouri et al. (2022) [27] investigated the prescription trends of PPIs in infants in Norway, Denmark, and Sweden. They found that the PPI dispensing levels increased significantly between 2007 and 2020, and Denmark had the highest rates. However, it was reported that after 2017, the PPI dispensing levels began to decline notably in Denmark [27]. In a study conducted to determine drug-related problems in newborns in Türkiye, Yalçın et al. (2022) determined that only 1 out of 412 newborns was prescribed pantoprazole, though it is not approved for infants under 1 year of age [28]. Despite the fact that they are not recommended by the global guidelines for childhood reflux and other GERD-related symptoms without confirmation, it can be seen that more children have been prescribed PPIs in recent years worldwide [3,12,29]. According to our results, the top five countries reporting the most PPI-related ADEs in infants are the United States, Great Britain, France, Spain, and Canada, respectively.

Although the FAERS database does not provide information about drug prescriptions directly, adverse event reports may provide indirect information about it. In our study, we showed that although there has been fluctuation since 2000, it is seen that the general trend tends to increase, and most of the adverse events were reported in 2016, similar to the PPI prescription worldwide discussed above. This might be associated with irrational drug prescribing and/or off-label PPI use. PPIs are often empirically prescribed for infantile reflux, functional dyspepsia, chronic cough, and asthma without documented associated gastroesophageal reflux disease (GERD). It has been observed that adverse event reporting has tended to decrease since 2016, as found by Lyamouri et al. (2022) in Denmark [27]. This trend is believed to have been significantly influenced by the guidelines published in 2015 by the NICE. The recommendations provided by the NICE are based on systematic reviews of the best available evidence, along with a clear evaluation of cost-effectiveness. Furthermore, the guidelines published in 2018 by NASPGHAN and ESPGHAN are also considered to have played a crucial role in reducing the unnecessary use of PPIs in neonates and infants in recent years.

### 3.2. Comparison of Each PPI

In a study by Dipasquale et al. (2022) [29], PPI-related ADR reports in children in the Italian Spontaneous Reporting System (SRS) database were investigated. In 70 reports, lansoprazole and esomeprazole were found to cause most of the adverse events in children equally [29]. Dipasquale et al. (2023) [30] continued their investigation about spontaneous adverse reaction reports associated with PPI prescription using the Italian SRS database. A total of 148 patients were included in this study, and the patients’ age range was 4–87 years. They found that most of the adverse drug reports belong to lansoprazole, followed by pantoprazole, esomeprazole, omeprazole, and rabeprazole [30].

In the current FAERS analysis, we detected that even though it is not approved for infants and children under the age of two, most of the ADEs were reported for lansoprazole, similar to the findings of Dipasquale et al. [30]. Lansoprazole is followed by omeprazole, esomeprazole, pantoprazole, and rabeprazole, respectively. We also analyzed the differences among PPI subclasses in terms of reported ADEs. For lansoprazole, vomiting was the most frequently reported ADE, followed by erythema. Similarly, vomiting was also the most commonly reported ADE for omeprazole, with hypertrichosis being the second most frequent. In the case of esomeprazole, pain was reported most often, followed by vomiting and edema (affecting the eye, eyelid, or lip), which occurred with equal frequency. Only a single report was available for rabeprazole, which involved a case of heart disease.

### 3.3. Comparison of Reporters and Outcomes of Each ADE

In terms of reporter type, Dipasquale et al. (2023) found that ADRs were reported mostly by healthcare professionals (physicians, pharmacists, and other healthcare professionals); only 4 out of 148 ADRs were reported by patients [30]. We also found that most of the ADEs were reported by healthcare professionals, followed by consumer reports.

Dipasquale et al. (2022) stated that 27.1% of the ADRs in the Italian SRS database were stated as “serious”. They found that most PPI-related adverse events were mild and reversible [29]. We observed that 69.6% of the cases were reported as “serious” and 30.4% as “non serious”; 3% of the cases had life-threatening issues, and 3% of the cases died.

### 3.4. Comparison of ADEs for Each Organ System

#### 3.4.1. Gastrointestinal Adverse Events

PPIs are usually well tolerated, though they might cause moderate side effects such as headache, nausea, constipation, and diarrhea in the short term [12]. The most common adverse events related to PPI use are reported as gastrointestinal symptoms (vomiting, constipation, and diarrhea) [3,14]. Dipasquale et al. (2022) indicated that 24% of PPI-related ADRs consisted of gastrointestinal symptoms [29]. In this study, we found that gastrointestinal adverse events (vomiting, diarrhea, constipation, abdominal pain, and pyloric stenosis) are the most reported (20.7%) adverse events.

Since regurgitation and vomiting are the most frequent symptoms of GERD in infants [31], it is important to determine whether the vomiting is PPI-related or a symptom of GERD. In a systematic review by King et al. (2025), they found that little or no difference was observed in the rate of vomiting episodes [6].

#### 3.4.2. Dermal Adverse Events

Dipasquale et al. (2022) found that 21.3% of PPI-related ADRs were dermal symptoms [29]. In this study, we found that dermal (erythema, rash, eczema, hypertrichosis, and hyperhidrosis) adverse events were the second most reported ADEs (14.7%), similar to Dipasquale et al. (2022)’s study [29].

In a study by Sandstrom et al. (2012) [32], the aim was to evaluate the steady-state pharmacokinetics and tolerability of repeated doses of intravenous esomeprazole in children. This multicenter, open-label study included 50 hospitalized patients aged 0 to 17 years who received once-daily intravenous esomeprazole sodium for injection over 4 days, with dosing based on age: 0.5 mg/kg (0–1 month), 1.0 mg/kg (1–11 months), 10 mg (1–5 years), 10 or 20 mg (6–11 years), and 20 or 40 mg (12–17 years). Adverse events were assessed throughout the study. As a result, erythema was only reported in three children aged 1–11 months [32].

Interestingly, we also found that PPI-associated hypertrichosis was reported in 19 patients. Elosua-González et al. (2018) [33] published a case report about omeprazole-induced hypertrichosis in two children in Spain. They concluded that omeprazole can increase prostaglandin E2 levels, which can stimulate hair growth and eventually cause hypertrichosis [34].

#### 3.4.3. Cardiopulmonary Adverse Events

A systematic review by King et al. (2025) [6] examined two studies to find out the connection between GERD and apnea, bradycardia, and desaturation events. They expressed that the two studies indicated little to no decrease in GERD-related events (bradycardia, apnea, desaturation, etc.) with PPI treatment [6]. In our study, we found that apnea and cardiopulmonary adverse events were reported in 7.8% of all patients.

#### 3.4.4. Thrombocytopenia, Hemorrhage, and Hematochezia

PPIs are also frequently used to prevent stress-induced ulcers and are sometimes advised to avoid upper gastrointestinal bleeding in patients who are admitted to the intensive care unit. Although it is uncommon, PPI medication is linked to a higher risk of thrombocytopenia in a few case reports. Watson et al. (2006) [34] investigated the relationship between pantoprazole use and thrombocytopenia using Naranjo algorithms (probable). They found that even though it is uncommon, it represents a potentially severe adverse effect [34]. Binnetoglu et al. (2015) also indicated that PPIs may cause thrombocytopenia, and patients who are treated with PPIs should be monitored more closely [35]. Similarly to the literature, we found that PPI-associated thrombocytopenia, hemorrhage, and hematochezia were reported in 6.9% of all patients.

#### 3.4.5. Nutrient Deficiencies

Chronic PPI use is linked with the risk of vitamin B12 and iron deficiencies, which may have a detrimental nutritional effect in infants [16,36,37]. Additionally, there are issues with calcium and magnesium absorption, which raises the risk of bone fractures [38]. In our study, PPI-associated vitamin–mineral deficiency is 1.7%. Six of the cases were zinc deficiency, one of the cases was vitamin B12 deficiency, and one of the cases was vitamin D deficiency.

#### 3.4.6. Infections

The use of PPIs has been implicated in an increased risk of infections, potentially due to alterations in the gut microbiota or direct immunomodulatory effects [13]. PPI-induced hypochlorhydria compromises the stomach’s bactericidal function, thereby predisposing individuals to enteric infections. Specifically in preterms, there is a clear association between PPI use and the subsequent development of necrotizing enterocolitis [20]. Long-term PPI use has been associated with a significantly elevated risk of Clostridium difficile, Campylobacter, or Salmonella gastroenteritis [39]. While studies in adults suggest a possible link between PPI therapy and a heightened susceptibility to intestinal infections, data on pediatric populations remain limited [40]. However, Lassalle et al. (2023) reported an increased incidence of severe infections—including gastrointestinal, lower respiratory tract, kidney/urinary tract, and nervous system infections caused by bacterial and viral pathogens—among children receiving long-term PPI treatment [13]. Furthermore, two meta-analyses identified a significant association between PPI use and an increased risk of Clostridium difficile infection in pediatric patients [41,42]. In our study, we observed a 1.3% prevalence of Clostridium difficile infection associated with PPI monotherapy.

#### 3.4.7. Allergy and Hypersensitivity Reactions

Emerging evidence suggests that the use of proton pump inhibitors (PPIs) may contribute to de novo type I allergic sensitizations to dietary components and orally administered drugs. Notably, the risk of developing food allergies in association with PPI therapy appears to be dose-dependent, increasing with the duration of treatment. Children prescribed PPIs for more than 60 days had a 52% higher likelihood of being diagnosed with a food allergy compared to those who received PPIs for 1 to 60 days. Maintaining a low gastric pH (1–3.5) is essential for the activation of gastric pepsin, as well as for triggering duodenal secretions and the subsequent release of pancreatic enzymes, which are crucial for digestion. By inhibiting gastric acid secretion, antacid medications may prevent the proper breakdown of food allergens, allowing them to remain intact and be absorbed, thereby increasing the likelihood of allergic sensitization [43]. Our study findings indicate that allergy (food and milk) and hypersensitivity reactions were reported in 12 cases (2.6%).

#### 3.4.8. Congenital Anomaly

Upon reviewing the case reports, it was found that congenital anomalies were reported in 19 cases. According to a study by Pasternak et al. (2010) [44], of 840.968 live births, 5082 had exposure to PPIs between 4 weeks before conception and the end of the first trimester of pregnancy. Among these, 174 infants (3.4%) were born with major birth defects, compared to 21.811 (2.6%) in the group whose mothers were not exposed to PPIs. This study concluded that exposure to PPIs during the first trimester was not significantly linked to an increased risk of major birth defects [44]. The study by Peron et al. (2023) [45] presents a meta-analysis examining the risk of major congenital malformations (MCMs) following PPI exposure during the first trimester of pregnancy. Analyzing data from 5618 pregnancies exposed to PPIs, this study found first trimester exposure was not linked to a significant increase in the risk of MCMs, based on the available scientific literature [45]. In a cohort study by Choi et al. (2023) [46], involving 2.696.216 pregnancies in South Korea between 2011 and 2019, the use of PPIs during the first trimester was not found to significantly increase the risk of major congenital malformations, congenital heart defects, cleft palate, hydrocephalus, or hypospadias. Additionally, sibling-controlled analyses indicated that PPIs were not likely to act as a major teratogen [46].

### 3.5. Medication Dosing or Administration Errors

In addition to adverse events, we found some useful additional information from the ADE reports retrieved from FAERS. The analysis of reports indicates that 11.4% of the cases were submitted despite the absence of any adverse events. Among ADE reports, 10.8% involved medication errors, while 6.5% were associated with prescribing errors. Additionally, in 9.3% of the reports, the use of PPIs was deemed inappropriate based on the patient’s age, and 7.8% of cases indicated the off-label use of PPIs. This trend suggests that healthcare professionals may be increasingly aware of the potential adverse effects of PPIs in infants and pediatric populations, leading them to report medication and prescribing errors even in the absence of an observed adverse event.

Drug poisoning in children represents a serious and potentially life-threatening issue. While such incidents are largely avoidable, there is still a notable gap in public awareness regarding the safe handling and storage of medications, especially in keeping them out of reach of children. Matalova et al. (2023) [47] conducted a retrospective study to determine drug-induced intoxications in pediatrics. They reported only three cases (1.9%) of PPI poisoning among a cohort of 162 children between 2010 and 2019 [47]. According to the data obtained from FAERS, accidental exposure was reported in only 24 out of 464 patients (5.2%).

Another notable finding in the ADE reports is the occurrence of issues related to medication administration in pediatric patients. In the reports, 8.4% mentioned “Wrong technique in product usage process”, 6.7% indicated “Incorrect dose administered”, 2.2% reported “Product quality-substitution issues”, and 1.5% noted “Oral administration complications”. It is estimated that the expression “wrong technique” is a problem experienced during the application. In order to prevent similar problems, 30–60 min before feeding, if there is OG/NG, be careful not to block it by dissolving in water or soft foods (e.g., yogurt, jam, or applesauce) without mixing with milk or carbonated liquids [48]. Another, though less commonly preferred, method is rectal administration, which produces consistent increases in esophageal and gastric pH in infants with pathological GERD, comparable to those observed with oral dosing in a randomized pilot trial [49].

### 3.6. Strengths and Limitations

There are several strengths to consider in this study. First, we utilized the FAERS database, a freely accessible online resource that provides a vast collection of real-world data. This extensive dataset allows for the examination of the occurrence and potential association of PPIs in infants and children. Unlike smaller, single-center research databases or registries, FAERS encompasses a much broader dataset derived from a global population.

However, certain limitations should also be acknowledged. Since the data were extracted from the FAERS database, which consists of adverse event reports submitted by pharmaceutical companies, healthcare professionals, and consumers, the dataset primarily relies on spontaneous reporting. Although publicly accessible via the FAERS Public Dashboard, the system contains incomplete and potentially duplicate reports that require careful evaluation. Additionally, these reports reflect the observations and opinions of the reporters and are not externally validated. As formal causality assessments are lacking in the publicly available dataset, there is no definitive confirmation that the reported adverse events were directly caused by the suspected substance.

### 3.7. Recommendations

Enhancing drug safety in pediatrics begins with the identification and detailed characterization of suspected adverse drug reactions (ADRs) and/or ADEs [50].

We used 2D disproportionality analysis (RORs) to determine whether the detected ADE is related to the drug of interest. We prepared this study using the READUS-PV [51] guidelines to develop transparent reports on disproportionality analysis, ensuring the accurate interpretation of findings and their limitations. Additionally, the guidelines support this study’s design and conception, facilitate self-assessment and reproducibility by other researchers, and enable the planning of pharmaco-epidemiological research based on the results of disproportionality analysis that align with the READUS-PV standards. This approach aims to enhance transparency and reproducibility throughout the research process, laying down a solid foundation for scientific inquiry.

Considering that the most important factors in terms of toxicity are the dose, duration of exposure, and route of exposure [52], the FAERS database does not contain information on the drug’s dose, duration of exposure, or the onset time of the adverse event. This absence of data makes the assessment more challenging. While the FAERS database offers valuable real-world insights into PPI-related ADEs, it does not provide definitive evidence that these events are directly associated with the active pharmaceutical ingredient (API) or substance in question. The underlying cause may instead involve a different drug, an existing medical condition, or simply a temporal correlation. Therefore, causality assessment plays a crucial role in guiding clinical decision-making and improving pharmacovigilance strategies. Although the regulatory framework for causality evaluation in neonates aligns with that of other populations, establishing a clear causal relationship in this age group remains particularly challenging [9]. Previous studies have identified notable limitations in widely used causality assessment tools, including the WHO–Uppsala Monitoring Centre (WHO-UMC) system and the Naranjo algorithm, particularly in their ability to accurately establish drug–adverse event relationships in neonatal populations [53,54]. In contrast, Leopoldino et al. (2023) noted that the Du algorithm—an adaptation of the Naranjo algorithm tailored for neonatal populations [55]—demonstrates superior sensitivity in identifying definite ADRs, making it a more suitable tool for routine clinical use in neonatology [56].

In terms of other evaluative dimensions such as seriousness and severity, ADEs are classified as “serious” or “non-serious” based on the FDA guidelines. For assessing severity specifically, the Neonatal Adverse Event Severity Score (NAESS) has been developed and validated as a reliable tool [53,57]. Although the process of severity assessment can be time-consuming, it offers valuable insight into the clinical impact of ADRs, thereby contributing to a more nuanced understanding of drug safety in neonatal care. Also, Leopoldino et al. (2025) [50] investigated the reliability of the Hartwig scale and the Liverpool Avoidability Assessment Tool (LAAT) in the pediatric population. The Hartwig scale is a widely used tool for assessing the severity of ADRs and has been shown to have good reproducibility among different evaluators. On the other hand, the Liverpool Avoidability Assessment Tool (LAAT), designed to evaluate the likelihood of avoiding an ADR, particularly in pediatric populations, was found to have low reproducibility [50].

Alongside FAERS, different online pharmacovigilance databases (e.g., Eudravigilance (Europe), DAEN (Australia), Vigibase (WHO), etc.) can be used to conduct a comprehensive comparative analysis. Also, longitudinal follow-up studies are needed to assess the potential developmental defects caused by the long-term use of PPIs in infants during the growth and development phase.

In personalized treatment approaches, it is recommended to integrate AI-based clinical decision support systems into electronic health records to prevent and monitor ADEs [57].

## 4. Materials and Methods

### 4.1. Study Design

We performed an observational, cross-sectional study using the FAERS database. We hereby identified all PPI (lansoprazole, dexlansoprazole, rabeprazole, pantoprazole, esomeprazole, and omeprazole)-related ADE reports entered between 30 June 2000 and 31 December 2024 (latest updated version in FAERS at the time of data extraction, 29 January 2025, and performed by the first author). Recognizing the need for standardized and transparent reporting in disproportionality analyses, this study follows the recommendations of the REporting of A Disproportionality analysis for drUg Safety signal detection using individual case safety reports in PharmacoVigilance (READUS-PV) statement, which was specifically developed to improve the quality and interpretability of pharmacovigilance signal detection based on individual case safety reports [58,59,60]. Ethical approval was waived due to the nature of the study design.

### 4.2. Data Extraction from the FAERS Database

In the FAERS database, we searched for ADEs reported in neonates or infants associated with PPI exposure. To achieve this, we first selected the active pharmaceutical ingredient (API) involved (lansoprazole, dexlansoprazole, rabeprazole, pantoprazole, esomeprazole, or omeprazole). After this, we filtered the study population as neonates (0–1 month) and 1 month–2-year-old infants. The selected data were extracted from the FAERS database. Duplications were checked.

Descriptive statistics and number and percentage values for categorical variables are provided. The reporting odds ratio (RORs) is commonly used to assess disproportionality between cases and non-cases and is currently endorsed by various reporting organizations and the World Health Organization [61]. In this study, the ROR (2D disproportionality analyses) was utilized to compare the odds ratio of the top 5 most frequently reported ADEs among neonatal and infant monotherapy strategies involving proton pump inhibitors (PPIs) (Table 2). An interaction signal was detected when the RORs for PPI monotherapy exceeded that of other index groups and when the lower bound of the 95% confidence interval (CI) surpassed 1. All analyses were carried out in Microsoft Excel version 16.89 software.

### 4.3. Data Handling and Analysis

Because of occasionally missing age data in the FAERS database, the maximum weight limit was set at 12 kg. Weight information indicated as lb was converted to kg via the “1 lb = 0.45 kg” formula [62]. Cases with both missing age and weight information were removed. Also, there were only 2 ADEs related to dexlansoprazole, but the age was stated as 30 months, so they were also excluded.

The reporting trends (annual number), specific infant outcomes (ADE), as mentioned in the ADE reports, and the number of ADEs according to the countries were described using absolute numbers and percentages. The ADE reports were classified using the generic name of the active pharmaceutical ingredient (API) involved.

## 5. Conclusions

Proton pump inhibitors (PPIs) carry potential risks and should be used with caution. The balance between their benefits and possible harms must be carefully assessed, with the lowest effective dose administered for the shortest duration necessary. In the treatment of gastroesophageal reflux disease (GERD) in infants, non-pharmacological interventions, such as adjusting feeding practices and optimizing body positioning after feeding, should be prioritized. When pharmacological treatment is required, the child’s response should be regularly monitored, and the duration of PPI use should be minimized. It is also important to know the duration of exposure, probability, and severity in the evaluation of ADEs. The long-term effects of PPIs on young children are still being studied, so treatment should be managed carefully and kept as short as possible. Additionally, in patients with polypharmacy, comorbidities, or enteral feeding, special attention should be paid to drug interactions and incompatibilities. Nurses and caregivers should be educated on proper drug preparation and administration techniques. A multidisciplinary approach is essential, with clinical pharmacists involved throughout the process to ensure that every step, from medication preparation to monitoring, is carefully managed.

## Figures and Tables

**Table 1:** Additional information retrieved from FAERS.

Additional Information	Number of Reports	%
No Adverse Event	53	11.4
Medication Error	50	10.8
Product Administered to Patient of Inappropriate Age	43	9.3
Wrong Technique in Product Usage Process	39	8.4
Off-Label Use	36	7.8
Incorrect Dose Administered	31	6.7
Product Prescribing Error	30	6.5
Accidental Exposure	24	5.2
Product Quality-Substitution Issue	10	2.2
Oral Administration Complication	7	1.5

**Table 2:** Fourfold table for measure of disproportionality.

	Adverse Event of Interest	All Other Adverse Events	Total
Drug of interest	a	b	a + b
All other drugs	c	d	c + d
Total	a + c	b + d	a + b + c + d

Reporting odds ratio (RORs) = (a × d)/(b × c), 95% CI = eln(RORs) ± 1.96√(1/a + 1/b + 1/c + 1/d).

## Data Availability

Upon request, the raw data supporting the conclusion of this article will be made available by the authors, without undue reservation, but can also be retrieved in the supplemental file.

## Supplementary Materials

The following supporting information can be downloaded at: https://www.mdpi.com/article/10.3390/ph18050730/s1, supplemental material, excel file with all data points.
